# Interests and concerns regarding medical marijuana among chronic pain patients in Ohio: an online survey

**DOI:** 10.1186/s42238-021-00092-y

**Published:** 2021-08-16

**Authors:** Daniel Adams, Nana Ama Ofei-Tenkorang, Patrick Connell, Alexa Owens, Aaron Gothard, Dmitri Souza, Samer Narouze

**Affiliations:** 1 Western Reserve Hospital Center for Pain Medicine, 1900 23rd St, Cuyahoga Falls, OH 44223 USA; 2 Ohio University Heritage College of Osteopathic Medicine, Athens, OH USA; 3 Lake Erie College of Osteopathic Medicine, Erie, PA USA; 4 Biostats, Inc., East Canton, USA

**Keywords:** Medical cannabis, Chronic pain, Patient attitudes, Ohio

## Abstract

**Background:**

Since the legalization of medical marijuana (MMJ) in Ohio in 2018, many chronic pain (CP) patients have become interested in it as an alternative or adjunct to prescription opioids. This has not only created a need for pain management specialists to learn about this potential indication for MMJ but also for them to have more detailed knowledge of patient attitudes and willingness to comply with providers’ recommendations regarding its safe use with other pain medications. For this purpose, we surveyed CP patients in a region severely affected by the opioid crisis in order to provide better education, formulate treatment plans, and develop clinical policies.

**Methods:**

We designed and administered the Medical Marijuana Interest Questionnaire (MMIQ) online to patients of the Western Reserve Hospital Center for Pain Medicine (CPM) with a diagnosis of CP who were not yet using MMJ. Questions addressed demographic and clinical characteristics, willingness to consider MMJ, and compliance with treatment plans and concerns. We then carried out a statistical analysis including Pearson chi-square, Spearman’s rho and Kendall’s tau tests to measure associations between variables to identify factors that may influence willingness to use MMJ.

**Results:**

After sending 1047 email invitations to complete the MMIQ, 242 (23.1%) completed questionnaires were returned. The average age range of all respondents was 51-60 years, 171 (70.7%) were female and 147 (60.7%) were current opioid users. The 204 (84.3%) respondents who were willing to consider using MMJ were given access to the entire questionnaire. Of these, 138 (67.6%) reported wanting to use less opioids after starting MMJ and 191 (93.6%) were amenable to following their pain specialists’ recommendations about using MMJ concurrently with opioids. Their greatest concern on a 0-5 scale was affordability (2.98) and there was a statistically significant negative correlation between older age and preference for inhaled forms (*p* = 0.023).

**Conclusion:**

The MMIQ was successful in eliciting important data regarding patients’ attitudes about MMJ for opioid titration and potential compliance. Our study was limited by being administered online rather than in-person, which skewed the demographic makeup of the sample. The MMIQ can be used to study similar populations or adapted to patients already using MMJ. Similar surveys of MMJ-experienced patients could be combined with chart reviews to study the success of these products for pain control and opioid substitution.

## Background

It has been estimated that chronic pain (CP) is one of the leading reasons for patients to seek medical care with 20% of Americans suffering from this group of conditions. Risk factors include female gender, low socioeconomic status, living in a rural area, and disability (Interagency Pain Research Coordinating Committee, 2016). Unfortunately, at least one-fifth of these patients continue to rely on opioid analgesics despite resulting complications and evidence against their use for most CP conditions (Yasaei et al., 2021). Pain specialists following guidelines for minimizing opioid use incorporate multimodal treatment models which include procedures, behavioral approaches, and coordinated care with psychologists, physical therapists, chiropractors, and occupational therapists (Chou et al., 2016). Even with these extensive approaches, however, the refractory nature of both opioid dependence and chronic pain is leading many to consider less conventional approaches such as medical marijuana (MMJ).

As of 2021, MMJ is legal in two-thirds of the USA and there is widespread interest in its potential to decrease the opioid burden of CP patients (Boehnke et al., 2019). However, a critical disparity has arisen between greater availability of MMJ and the lack of clear evidence for its effectiveness and safety (Bigand et al., 2019; Nugent et al., 2017). As a result, pain specialists and other medical providers are often faced with the dilemma of responding to patients’ questions without the high level of confidence they have with conventional medications. In addition to educating themselves, they can partially address this knowledge gap by gathering information from patients in order to determine the best candidates for MMJ therapy, select educational materials, and prepare institutional protocols. Though there have been some surveys seeking to characterize other populations of MMJ and recreational marijuana users, little data have been collected to date specifically on the attitudes of CP patients (Takakuwa & Sulak, 2020).

We surveyed patients with CP at the Western Reserve Hospital Center for Pain Medicine (CPM) who had not yet tried MMJ regarding their interest in it. Though some of our patients had already begun using MMJ and a study of this population could yield useful data, we chose to study MMJ-naïve patients for several reasons. First, they represented a sufficiently large sample from which to draw statistically significant conclusions, whereas only several dozen patients had experience with it. We decided that this much smaller group would be better studied retrospectively and presented with descriptive rather than statistical methods. Another factor that led to this decision was the implausibility of formulating the same questions for both MMJ-naïve and MMJ-experienced patients. Therefore, we decided to research both groups separately but to study the larger group first, as the need to address their educational and clinical needs was more relevant to our daily practice.

The survey concerned three main lines of inquiry. The first was patients’ interest in MMJ for the purpose of reducing opioids, a class of drugs with significant adverse events, addiction potential, and long-term health risks (Abdel Shaheed et al., 2016). Though patients are often reluctant to accept alternatives to opioid therapy, our anecdotal experience was that patients were very curious about MMJ as a replacement and often brought up this topic in office visits. Our second goal was to assess compliance with recommendations of dosing and routes of administration. This was of particular interest to us because current MMJ laws in Ohio do not allow MMJ-certified providers to specify doses, cannabinoid ratios, or routes of administration as with other prescribed drugs (The Ohio Legislature, 2018). Instead, these factors are determined by the dispensary staff while providers can only see what products were actually bought weeks later in the prescription drug monitoring report (PDMP). This policy may contribute to the view of many Ohio physicians that MMJ laws are too lenient and limit their willingness to become certified providers of MMJ cards (Lombardi et al., 2020). The third area of inquiry regarded patients’ concerns about using MMJ, as knowing about them could inform risk-benefit discussions involving safety and affordability.

In addition to gathering information to further our clinicians’ goals of opioid reduction, safety, and education, we wanted to contribute to the growing literature on attitudes toward MMJ in various geographic locations (Rochford et al., 2019; Lintzeris et al., 2020; Azcarate et al., 2020). Indeed, the population discussed here was particularly relevant for two reasons. First, MMJ is relatively new in the state of Ohio, having been legalized in 2018 for approved conditions (see Appendix 1). Since then, patients were exposed to frequent media coverage of MMJ developments from initial legislation to dispensary openings. Secondly, the reduction of opioids is of particular interest to them as residents of Summit County, an epicenter of the opioid crisis and litigation with prescription opioid manufacturers covered in the national media (Cooper et al., 2019).

## Methods

Our search of the PubMed database resulted in 44 articles related to surveys of MMJ for pain conditions. Not finding a validated survey we felt was applicable to our specific goals, we developed the Medical Marijuana Interest Questionnaire (MMIQ) based on selected surveys from our search, patient questions, the approved indications for MMJ in Ohio, and our prior experience in survey research (Lombardi et al., 2020; Rochford et al., 2019; Piper et al., 2017a; Narouze et al., 2020). This resulted in 25 questions divided into 5 categories: demographics, clinical profile, expectations, adherence, and concerns. The format for each question was chosen based on ease of comprehension by respondents and applicability of statistical methods. The MMIQ was then entered into the Google Forms online survey platform and piloted with approximately 15 patients, laypersons, and clinical staff who provided feedback prior to the final version (Appendix 2).

The first section of the MMIQ queried independent variables including demographic data (gender, ethnicity, education level, age) followed by current opioid use and willingness to try MMJ. Patients responding negatively to this last question were not allowed to answer further questions, but their answers to this section were recorded. The second section (beginning with independent variables) started by asking what complaints they might seek MMJ treatment for. We divided these into primary pain complaints (back pain, arthritis, neck pain, neuropathy, fibromyalgia, pain from past surgery, headache, other) and secondary psychosocial complaints (anxiety, depression, attention disorder, sleep, using less opioids, muscle spasm, PTSD, nausea, appetite, other). Since many of our patients suffer from multiple painful conditions, we gave them the option of selecting more than one complaint so that the results would more accurately reflect the prevalence of each in this population. The next questions were average Numeric Pain Rating Scale (NPRS) score over the previous week, expectation of percentage pain relief from MMJ (< 25%, 25-50%, 50-75%, 75-100%, not sure), preferred forms (edibles, pills, topical, sublingual drops, inhaled), and acceptable monthly expenditure (< $50, $50-100, $100-200, $200-300, $300-400, > $400). Adherence was assessed by querying patients’ inclinations to: use the form and dosage suggested by their provider, attend monthly visits for safety monitoring, take less opioids and benzodiazepines while using MMJ, use MMJ even if that meant they could not take opioids, and participate in research on MMJ. For the final questions on concerns about MMJ use, we used a 0-5 point scale (0 = “not concerned at all,” 5 = “wouldn’t use MMJ because of this”). Types of concerns included overdose, addiction, disapproval of friends and family, affordability, side effects/drug interactions, and driving safety.

Prior to administering the survey, we obtained approval of the Western Reserve Hospital Research Committee and waiver of IRB review from the Lake Erie College of Osteopathic Medicine Institutional Review Board (Appendix 3). This waiver was granted on the basis that (1) no direct or indirect patient identifiers were given by respondents, (2) clinical staff (including health providers) had no knowledge of their patients’ responses, and (3) the Google Forms platform was not enabled to collect any IP addresses from respondents. Per IRB policy, this also granted waiver of written informed consent.

Our initial protocol involved administering the MMIQ in-person by clinical staff on touchscreen devices, but a suspension of office visits due to the COVID-19 pandemic led us to distribute the survey via email instead. Using the eClinicalWorks® electronic medical record search engine of patients at CPM, we generated a list of 1047 patients based on the following criteria: > 18 years of age, diagnosis of chronic pain (ICD-10 code G89.4), and seen in the last 6 months. We emailed them a brief explanation of the survey with a link to the Google Forms page. This invitation directed them not to start the MMIQ if they had already tried MMJ or were currently using it. All responses were received between April 7, 2020, and May 20, 2020. We had determined previously that 200 responses were needed for statistical estimation of categorical percentages within a margin of error of +/− 7%. The data were then imported into a spreadsheet for analysis by a biostatistician.

### Statistical analysis

Survey data were imported into SPSSv25.0 software (IBM Corp., Armonk, NY). Cohort summaries were presented using frequencies and percentages for categorical data and mean, standard deviations, and minimum/maximum values for numeric data. Associations that were examined included (1) willingness to consider MMJ with demographics and opioid use, (2) opioid use with NPRS score, (3) opioid use with concerns, (4) age with concern of disapproval, (5) age with concern regarding driving safety, (6) age with preferred form, (7) NPRS score with type of pain, and (8) NPRS score with expected percentage relief. Pearson chi-square tests were performed to measure the association between nominally measured data. Kendall’s tau tests were performed to examine the relationship between dichotomous nominal data and ordinally scaled metrics. Spearman’s rho values were determined to measure the association between ordinally scaled data and tested for equivalence to zero. All statistical tests were two-sided with *p* < 0.05 considered statistically significant.

## Results

### Demographics

Of the 1047 survey invitations sent, 242 (23.3%) patients responded. In brief, 70.7% (*n* = 171) of all responders identified as female, 90.5% (*n* = 219) as Caucasian/White and 29.7% (*n* = 72) reported at least some college education. The mean of the age ranges was 51-60 and 60.7% reported taking opioids as part of their current treatment regimen. Two hundred four (84.3%) reported they would consider trying MMJ (Table 1). In comparing all invitees (*n* = 1047), respondents (*n* = 242), and respondents willing to consider MMJ (*n* = 204), the latter group had less higher education and the group of all invitees skewed toward a higher average age (63.5 years) than respondents. Otherwise, there were no significant differences between groups (Table 2).

**Table 1:** Responses to questions 5 and 6: Current opioid use and willingness to consider medical marijuana among all respondents (*n* = 242)

	*N* (%)
**Opioid use**	
Yes	147 (60.7%)
No	95 (39.3%)
**Would consider MMJ?**	
Yes	204 (84.3%)
No	38 (15.7%)

**Table 2:** Responses to questions 1-4: Demographic characteristics of all chronic pain patients sent invitations to complete the survey. Columns indicate those considering medical marijuana, all respondents and all invitees

	Consider MMJ (***n*** = 204) *N* (%)	Respondents (n = 242) *N* (%)	Invitees^**a**^ (n = 1047) *N* (%)
**Gender**			
Male	65 (31.9%)	71 (29.3%)	330 (31.5%)
Female	139 (81.3%)	171 (70.7%)	717 (68.5%)
**Age (years)**			
18-30	2 (1%)	2 (0.8%)	4 (0.3%)
31-40	12 (5.9%)	19 (7.9%)	47 (4.5%)
41-50	36 (17.6%)	43 (17.8%)	134 (12.8%)
51-60	66 (32.4%)	78 (32.2%)	246 (23.5%)
61-70	58 (28.4%)	66 (27.3%)	298 (28.6%)
71-80	23 (11.3%)	26 (10.7%)	197 (18.8%)
81 +	7 (3.4%)	8 (3.3%)	121 (11.5%)
Modal age category	51-60	51-60	61-70
**Ethnicity**			
African American	15 (7.3%)	18 (7.4%)	
Native American	2 (1%)	2 (0.8%)	
Asian	1 (0.5%)	1 (0.4%)	
Caucasian/White	186 (91.2%)	219 (90.5%)	
Hispanic/Latino (a)	0 (0)	1 (0.4%)	
Other	0 (0)	1 (0.4%)	
**Education level**			
< High school	3 (1.5%)	3 (1.2%)	
High school grad.	60 (29.4%)	19 (7.9%)	
Some college	63 (30.9%)	69 (28.5%)	
College grad.	60 (29.4%)	70 (28.8%)	
Graduate school	18 (8.8%)	81 (33.5%)	

^a^Age in this column was extracted from the electronic medical record and converted to the ranges used by respondents

### Clinical profile and expectations

**Fig. 1:**
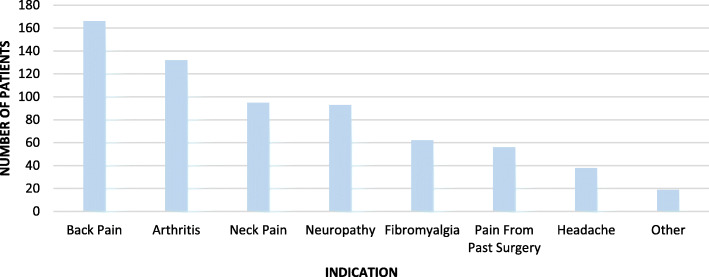
Responses to question 7: Primary complaints/indications for medical marijuana among those willing to consider it (*n* = 204). Respondents were allowed multiple selections

**Fig. 2:**
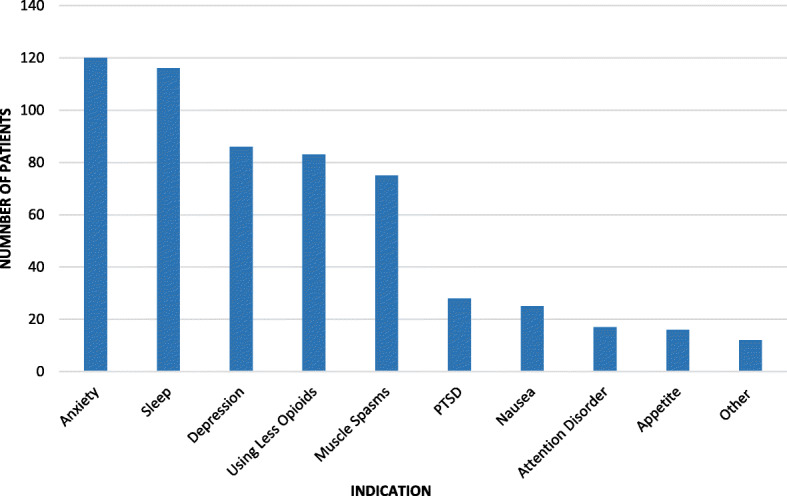
Responses to question 8: Secondary complaints/indications for medical marijuana among those willing to consider it (*n* = 204). Respondents were allowed multiple selections

**Fig. 3:**
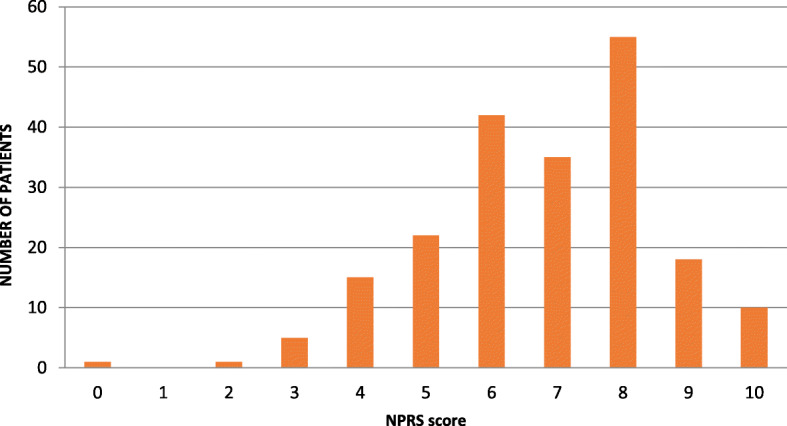
Responses to question 9: Average reported pain (Numeric Pain Rating Scale score) over the previous week among those willing to consider medical marijuana (*n* = 204). Mean = 6.79/10, SD = 1.76

**Fig. 4:**
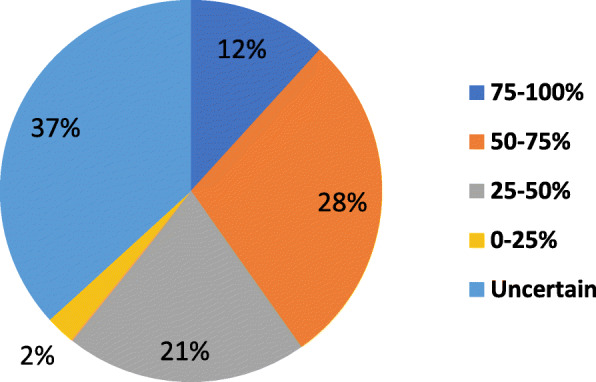
Responses to question 10: Expected percentage of pain relief (reported as ranges) from medical marijuana among those willing to consider it (*n* = 204)

**Fig. 5:**
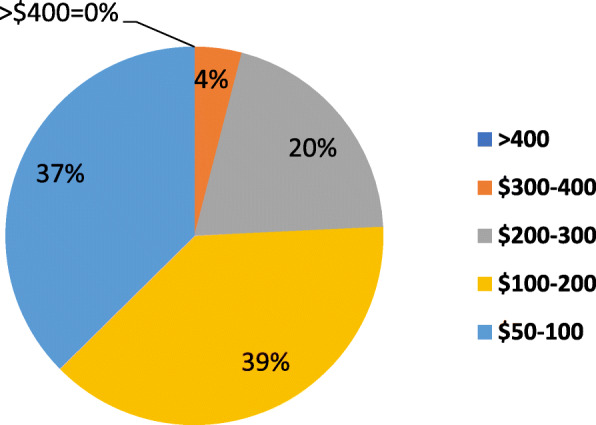
Responses to question 12: Expected monthly cost of medical marijuana among those willing to consider it (*n* = 204)

**Fig. 6:**
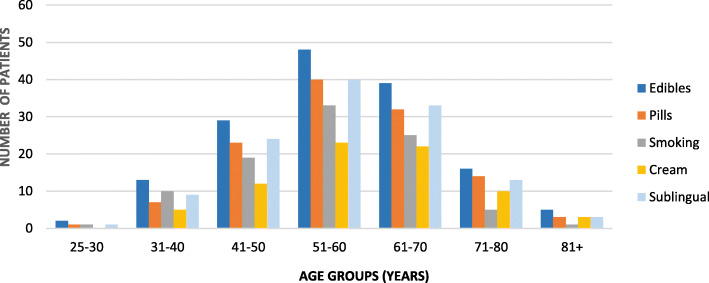
Responses to question 11 by age group: Preferred form of medical marijuana by age range among those willing to consider it (*n* = 204). Respondents were allowed multiple selections

The type and prevalence of primary and secondary complaints are shown in Figs. 1 and 2, respectively (multiple selections were allowed). The average NPRS score over the previous week for all respondents was 6.79/10 (Fig. 3). The percentage of expected pain relief was reported in the following ranges: 11.7% (*n* = 24) for 75-100% relief, 28.8% (*n* = 58) for 50-75%, 20.5% (*n* = 42) for 25-50% relief, 2.4% (*n* = 5) for 0-25%, and 36.6% (*n* = 75) reported being uncertain (Fig. 4). The monthly acceptable expenditure on MMJ was only reported by 198 respondents (6 were apparently not recorded by Google Forms): 37.2% (*n* = 74) preferred spending below $50, 38.7% (*n* = 76) were willing to spend $50-100, 20.2% (*n* = 40) to spend $100-200, 4.0% (*n* = 8) to spend $200-400 while no respondents wanted to spend more than $400 (Fig. 5). Patients were allowed multiple selections to indicate their preferences for routes of administration/forms of MMJ, which resulted in 567 selections: edibles (74.6%, *n* = 153), sublingual drops (60.5%, *n* = 124), pills (58.5%, *n* = 120), inhaled (46.3%, *n* = 95), and topical (36.6%, *n* = 75). Figure 6 shows preferred routes of administration by age group.

### Adherence

A total of 93.6% (*n* = 191) agreed to follow recommended forms and dosages while the other 6.4% (*n* = 13) responded “maybe.” In total, 93.6% (*n* = 191) were ready to attend monthly visits for monitoring safety, 5.4% (*n* = 11) answered “maybe” and 1% (*n* = 2) reported unwillingness. In patients taking opioids, 68.1% (*n* = 139) were willing to take less with concomitant MMJ, 29.4% (*n* = 60) answered “maybe” and 2.5% (*n* = 5) answered “no.” Of those using benzodiazepines (*n* = 56), 75.0% (*n* = 42) were willing to wean off these after starting MMJ, 19.6% (*n* = 11) answered “maybe” and 5.4% (*n* = 3) answered “no.” Thus, a greater proportion of benzodiazepine users reported interest in decreasing these medications versus opioid users (75% vs. 67.8%). A total of 71.6% (*n* = 146) of respondents expressed interest in participating in research.

### Concerns

**Fig. 7:**
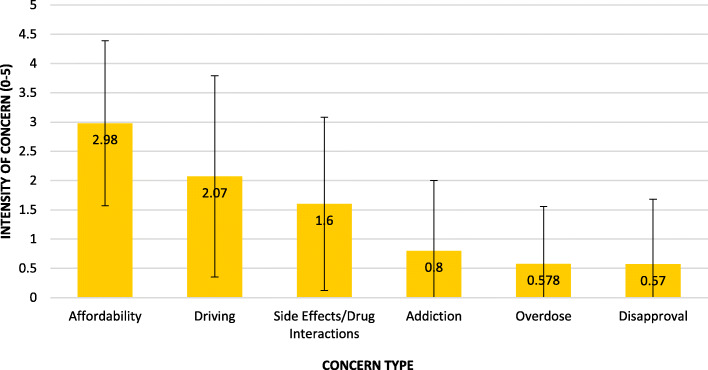
Responses to questions 19-25: Concerns about medical marijuana among those willing to consider it (*n* = 204). Respondents were asked to rate these on a 0-5 point scale (0 = “not concerned at all” to 5 = “wouldn't use MMJ because of this”). Columns represent the mean levels of concerns +/− 1 SD error bars

The average level of each concern is shown in Fig. 7. Affordability was the greatest with an average score of 2.98/5. However, only 15% (*n* = 31) rated this as a 5. Respondents were least concerned about the disapproval of family and friends, rating this on average as 0.57/5.

### Associations

Willingness to consider MMJ was marginally higher among men (91.5% vs. 81.3% of women, *p* = 0.053) and opioid users (87.8% vs. 78.9% of non-opioid users, *p* = 0.073). There was no association between willingness and age (Table 3 in Appendix 4). There was a statistically significant negative correlation between age and preference for inhaled forms of MMJ (tau = −0.144, *p* = 0.023) with those over the age of 70 being much less likely to choose this route of administration (Table 4 in Appendix 4). Older patients also showed a marginally significant preference for topical forms (tau = 0.115, *p* = 0.069). There were no significant associations between the following: (1) Average NPRS score over the previous week and expected relief [Table 5 in Appendix 4]; (2) opioid use and pain score or concerns [Tables 6 and 7 in Appendix 4]; (3) age and concern for disapproval or age and driving safety [Table 8 in Appendix 4].

Though not relevant to the goals of this survey, we should note that statistical significance can be assigned to the slightly higher pain scores of those with arthritis (7.0/10, *p* = 0.044) and fibromyalgia (7.2/10, *p* = 0.032) than average (6.79/10). A linear regression with pain modeled by fibromyalgia indication and female gender showed an insignificant contribution by the female gender factor (adding 0.227 to the pain score, *p* = 0.402). Headache sufferers reported slightly more pain as well, but this was only marginally significant (7.3/10, *p* = 0.068).

## Discussion

Even for pain specialists interested in MMJ as a treatment option, uncertainty about regulations, possible indications, pharmacology, compliance, or safety can prevent them from recommending it to patients. In a survey of 348 Ohio physicians’ attitudes toward the state’s medical cannabis program, Lombardi et al. (2020) found that roughly 40% felt that the program was too lenient and that approximately 60% were unlikely to recommend it as a result. In another survey of physicians, Narouze et al. showed that even those with favorable attitudes toward cannabis did not feel they had enough knowledge to recommend (Narouze et al., 2020). Given the inability of Ohio physicians to prescribe specific doses and forms, few FDA-approved cannabis-derived medications and limited evidence of effectiveness and safety, it is no surprise that uncertainty exists in addressing patient queries and forming clinical policies. While legal and medical support for clinicians may develop slowly, we hypothesized that surveying patients could provide useful insights in the short term, especially if applied to patient education. Indeed, studies have demonstrated that effective counseling may influence patients’ willingness to use MMJ, how they take it, and instill realistic expectations of its benefits (Parihar et al., 2020).

### Summary of principal findings

The chief objective of this study was to assess the attitudes of CP patients at our clinic toward MMJ, especially as a replacement for opioid therapy. The results describe a population mostly willing to try MMJ, use less opioids and benzodiazepines, comply with safety requirements, and use MMJ as advised. We also found that our patients perceived MMJ as a relatively safe and acceptable therapy compared to opioids. Except for one minor error in reporting the full number of responses, Google Forms proved a reliable platform to conduct the survey.

### Limitations

The main weaknesses of our study are the result of it being administered online rather than in our clinic as originally planned. As a result of this change, respondents may have held stronger or more positive opinions about MMJ than those who did not participate. This could have created bias favoring a hypothesis that patients are more interested in MMJ or have shifted results toward those with internet access, education, time to respond, or ability to spend money on MMJ. In fact, a higher proportion of respondents reported as Caucasian (90.5%) and having completed college (62.3%) than Summit County residents overall (78.2% Caucasian and 35.8% with college degrees) according to U.S. Census data (United States Census, 2020). In addition, the older age of all invitees than actual respondents is worthy of mention, as it may indicate that some of the email addresses for elderly patients were actually those of caretakers, or that those patients were not as responsive to this type of communication. On the other hand, online administration may have allowed patients to feel more anonymity than during office visits, where perceived disapproval or observation by clinical staff could have stifled candidness.

Another weakness of our study was the relatively small sample. A greater response rate may have resulted in stronger correlations or granularity in terms of subgroup characteristics. For example, male gender and lower education level may be more associated with MMJ interest than we were able to demonstrate. A larger sample might also have allowed us to validate our questions through psychometric analysis and draw stronger comparisons with similar surveys of other populations. It should be noted that the high percentage of women respondents (70.6%) is consistent with the gender makeup of our clinic and does not, therefore, suggest an error with study design. Nonetheless, studies of other populations may demonstrate less of a disparity, since 22.1% of women in the general population have CP compared to 18.6% of men (Dahlhamer et al., 2018).

In drafting this article, another weakness of the questionnaire was brought to our attention. This was that those who responded negatively to question #6, “Would you consider taking medical marijuana (MMJ) for your pain?” might have contributed to the data on concerns. This could have been remedied by including the section on concerns earlier in the survey before these respondents were screened out of answering further questions.

Finally, we would be remiss in not acknowledging a limitation inherent in all survey research: responses representing opinions and attitudes do not necessarily predict actual behavior.

### Meaning and implications for patient education

Our data suggest that education in this population needs to address several issues related to patient concerns about safety. Most significantly, the average rating of concern for driving under the influence of cannabis (DUIC) on the 0-5 scale was only 2.07 with 10% (*n* = 21) reporting a 5 (Fig. 7). However, some research indicates that this is a greater danger than they may be aware of. One study showed that over half of those surveyed reported driving within 2 h of taking MMJ and there have been correlations between driving deaths and positive THC tests in Washington and Colorado (Bonar et al., 2019; NIDA, 2008). Therefore, patients should probably be made aware of local laws regarding DUIC, as 15 states have legislation specifically addressing this issue. Clinicians may also choose to incorporate rules about DUIC into pain treatment contracts. Another possibly under-rated concern category was “side effects and drug interactions,” which was on average 1.6/5 with only 7 patients (3%) reporting a 5/5. This could reflect anecdotal experiences of recreational use rather than how MMJ is used in the setting of chronic conditions, co-morbidities and polypharmacy. Also, it should be noted here that older patients’ aversion to inhaled MMJ products may actually be beneficial from a safety standpoint, as these have been found to be less safe than other routes of administration (Fischer et al., 2017).

Our results showed that expenditure on MMJ and related products may also have important implications for patient education and long-term compliance with treatment. Specifically, the data show that patients’ expectations of out-of-pocket expenses are lower than recent market prices. The typical cost of obtaining an MMJ card in Ohio is around $200 and the average receipt total for marijuana plant-containing products at a dispensary is approximately $140 (Ohio Board of Pharmacy, 2020). With additional supplies such as empty capsules, de-carboxylizers, and safe storage devices, the initial investment can easily reach above $500. Given that 36.6% of patients were uncertain of how much pain relief they would receive from MMJ and 75.9% reported not wanting to spend more than $100 per month, knowing the costs of MMJ products at local dispensaries may be helpful to patients considering this form of therapy. We should note the difficulty of comparing our respondents’ concerns to those in other surveys, since most of those query patients’ experience with MMJ rather than their perception of it prior to use. However, our patients’ concern for price of MMJ and related costs appears to be well-founded and consistent with other surveys. Greater-than-expected expenditure on MMJ was shown to be a discouraging factor for many respondents in a high-powered survey of patients in New England (Piper et al., 2017b).

Aside from safety and cost, the lack of a statistically significant association between concerns and clinical characteristics with demographic factors suggests that educational materials may not necessarily need to be tailored to diagnosis, age, gender, or opioid use. However, the limited size of our sample and its relatively homogenous demographics limits the applicability of this statement to our respondents. It is likely that knowledge of factors such as health literacy and ethnicity would enhance educational approaches in other populations.

### Future research

This version of the MMIQ may be useful in future studies or clinical applications. We suggest that it could be administered individually prior to discussions of MMJ treatment to provide patient-specific counseling. It might also be validated and used to assess the differences between populations (clinical, geographic, etc.). For instance, there could be significant differences between chronic pain patients and those from other clinical settings who are less accustomed to complying with contracts, pill counts, drug screens, etc.

In addition to these other uses of the MMIQ, more surveys of CP patients who have already tried MMJ will be useful in assessing effectiveness and safety given the lack of prospective studies to date. As stated above, it is our intention to perform a retrospective chart review of MMJ-experienced CP patients, perhaps in conjunction with a survey to assess their satisfaction with MMJ, costs, pain relief, possible legal or occupational problems, and side effects. Our anecdotal experience is that some patients often try MMJ but return to opioid therapy due to its higher cost and lack of effectiveness, so such a study could elucidate the actual prevalence and reasons for these patients’ decisions.

## Conclusion

While developing the MMIQ, we identified areas of inquiry that would be useful in evaluating patient attitudes toward using MMJ for opioid replacement. The survey was also designed to gather information that pain specialists need to create educational materials and assess compliance. Although the collected data showed a willingness to consider MMJ and comply with safety regulations, it also suggested that patient expectations of pain reduction and expenditure may not be realistic given current clinical evidence and MMJ prices in Ohio. Though there may not be a robust evidence base yet about MMJ as an opioid sparing tool, we feel that these results are the basis for providing more useful and patient-centered education to this population.

## Data Availability

The data used for this study are available from the corresponding author by request.

## Appendix 1

### Qualifying conditions for medical marijuana in Ohio

**Table:**

▪ AIDS ▪ Amyotrophic lateral sclerosis ▪ Alzheimer’s disease ▪ Cachexia ▪ Cancer ▪ Chronic traumatic encephalopathy ▪ Crohn’s disease ▪ Epilepsy or another seizure disorder ▪ Fibromyalgia ▪ Glaucoma ▪ Hepatitis C	▪ Inflammatory bowel disease ▪ Multiple sclerosis ▪ Pain: chronic and severe or intractable ▪ Parkinson’s disease ▪ Positive status for HIV ▪ Post-traumatic stress disorder ▪ Pickle cell anemia ▪ Spinal cord disease or injury ▪ Tourette syndrome ▪ Traumatic brain injury ▪ Ulcerative colitis

## Appendix 2

### Medical Marijuana Interest Questionnaire

**Table:**

**#**	**Question**	**Possible answers**
**1**	What is your gender?	Female, male, other/non-binary
**2**	How old are you?	18-24, 25-30, 31-40, 41-50, 51-60, 61-70, 71-80, 81, or older
**3**	What is your ethnicity?	African American, American Indian/Native American, Asian, Caucasian/white, Hispanic/Latino(a), Pacific Islander, Other
**4**	What is your level of education?	High school, some college, college graduate, grad. school
**5**	Do you take opioids for your pain? (Percocet, Norco, Dilaudid, tramadol, hydrocodone, and oxycodone)	Yes, no
**6**	Would you consider taking medical marijuana (MMJ) for your pain?	Yes (leads respondent to #7), no (ends participation)
**7**	What pain problem would you want to take it for? (check all that apply)	Back pain, neck pain, headache, neuropathy, fibromyalgia, pain from a past surgery, arthritis, other
**8**	What other problems would you want it to help with? (check all that apply)	Attention disorder (ADD or ADHD), anxiety, appetite, depression, muscle spasms, nausea, PTSD, seizures, sleep, using less opioids, other
**9**	What was your average pain score over the last week?	0, 1, 2, 3, 4, 5, 6, 7, 8, 9, 10
**10**	How much pain relief would you expect to get from MMJ?	0-25%, 25-50%, 50-75%, 75-100%
**11**	What forms of MMJ would you consider using? (check all that apply)	Edibles, pills, drops under tongue, inhalation, skin cream/ointment
**12**	How much would you be prepared to spend on it each month?	Under $50, $50-100, $100-200, $200-400, over $400
**13**	Would you agree to use only the form and dose suggested by your doctor?	Yes, no, maybe
**14**	Would you be willing to attend monthly office visits for the first few months to monitor safety?	Yes, no, maybe
**15**	Would you be willing to take less opioids or stop taking them while using MMJ?	Yes, no, maybe
**16**	Would you be willing to take less benzodiazepines or stop them while using MMJ?	Yes, no, maybe, I don’t use benzodiazepines
**17**	Would you use MMJ even if it meant that you could not be prescribed opioids anymore?	Yes, no, maybe
**18**	Would you be willing to participate in research on MMJ?	Yes, no, maybe
**19**	How concerned are you about an overdose from MMJ?	
**20**	How concerned are you about becoming addicted to MMJ?	
**21**	How concerned are you about the disapproval of family, friends, etc.?	
**22**	How concerned are you about the MMJ you buy being used by family, friends, etc.?	
**23**	How concerned are you about side effects or drug interactions?	
**24**	How concerned are you about driving safely after using MMJ?	
**25**	How concerned are you about testing positive on an employee drug screen?	
Possible answers for #19-25: 0 = not concerned at all; 1, 2, 3, 4, 5 = wouldn’t use MMJ because of this		

## Appendix 3

### Rationale for exemption of IRB review

“Thank you for submitting the above-captioned research protocol to the LECOM Institutional Review Board. Research utilizing survey procedures with adults is exempt from the requirement for Institutional Review Board review and approval per the exemption at CFR 46. 104(d)(2) unless the data are recorded in such a manner that human subjects can be identified and any disclosure of subjects responses outside the research could place the subjects at risk of criminal or civil liability, or be damaging to their financial standing, employability, or reputation.

As described in your submission (which includes your original submission of October 31, 2019, and your email messages of November 25, 2019, and December 3, 2019, containing clarifications), you wish to (sic) patients of the Center for Pain Management regarding their interest in and concerns about the use of medical marijuana for pain control. The survey itself requests no direct or indirect identifiers. Patients will be asked to complete the survey on an iPad at their appointment and will return the iPad to a Center staff member after submission of the survey on Google Forms. You have represented that staff members will be unable to see any responses and that any surveys not successfully submitted by a subject will be disregarded. You have further represented that you will not enable the feature of Google Forms that collects IP addresses. Based on these protections of confidentiality, I have determined that the project is exempt from the requirement for IRB review and approval.

## Appendix 4

### Supplemental statistical tables

**Table 3:** Associations with medical marijuana consideration: gender, opioid use, age (*n* = 242)

	Consider MMJ?	
	Yes *N* (%)	No *N* (%)
**Gender**		
Male	65 (91.5%)	6 (8.5%)
Female	139 (81.3%)	32 (18.7%)
*p* = 0.053		
**Opioid use**		
Yes	129 (87.8%)	18 (12.2%)
No	75 (78.9%)	20 (21.1%)
*p* = 0.073		
**Age (years)**		
25-30	2 (100%)	0 (0.0%)
31-40	15 (78.9%)	4 (21.1%)
41-50	38 (88.4%)	5 (11.6%)
51-60	69 (88.5%)	9 (11.5%)
61-70	55 (83.3%)	11 (16.7%)
71-80	20 (76.9%)	6 (23.1%)
81 +	5 (62.5%)	3 (37.5%)
*p* = 0.151, Kendall’s tau = −0.084

**Table 4:** Associations between age and preferred forms of medical marijuana (*n* = 204)

Form of MMJ	Age (years)						
Edibles	25-30	31-40	41-50	51-60	61-70	71-80	81+
Yes	2 (100%)	13 (86.7%)	29 (76.3%)	48 (69.6%)	39 (70.9%)	16 (80.0%)	5 (100%)
No	0 (0.0%)	2 (13.3%)	9 (23.7%)	21 (30.4%)	16 (29.1%)	4 (20.0%)	0 (0.0%)
*p* = 0.783, Kendall’s tau = −0.018							
**Pills**	25-30	31-40	41-50	51-60	61-70	71-80	81+
Yes	1 (50.0%)	7 (46.7%)	23 (60.5%)	40 (58.0%)	32 (58.2%)	14 (70.0%)	3 (60.0%)
No	1 (50.0%)	8 (53.3%)	15 (39.5%)	29 (42.0%)	23 (41.8%)	6 (30.0%)	2 (40.0%)
*p* = 0.406, Kendall’s tau = 0.053							
**Smoking**	25-30	31-40	41-50	51-60	61-70	71-80	81+
Yes	1 (50.0%)	10 (66.7%)	19 (50.0%)	33 (47.8%)	25 (45.5%)	5 (25.0%)	1 (20.0%)
No	1 (50.0%)	5 (33.3%)	19 (50.0%)	36 (52.2%)	30 (54.5%)	15 (75.0%)	4 (80.0%)
^a^*p* = 0.023, Kendall’s tau = −0.144							
**Topical**	25-30	31-40	41-50	51-60	61-70	71-80	81+
Yes	0 (0.0%)	5 (33.3%)	12 (31.6%)	23 (33.3%)	22 (40.0%)	10 (50.0%)	3 (60.0%)
No	2 (100%)	10 (66.7%)	26 (68.4%)	46 (66.7%)	33 (60.0%)	10 (50.0%)	2 (40.0%)
*p* = 0.069, Kendall’s tau = 0.115							
**Sublingual**	25-30	31-40	41-50	51-60	61-70	71-80	81+
Yes	1 (50.0%)	9 (60.0%)	24 (63.2%)	40 (58.0%)	33 (60.0%)	13 (65.0%)	3 (60.0%)
No	1 (50.0%)	6 (40.0%)	14 (36.8%)	29 (42.0%)	22 (40.0%)	7 (35.0%)	2 (40.0%)
*p* = 0.910, Kendall’s tau = 0.007							

^a^Statistically significant finding

**Table 5:** Association between Numeric Pain Rating Scale score and expected percentage relief (*n* = 204)

	Pain score										
Relief %	0	1	2	3	4	5	6	7	8	9	10
0-25	0 (0.0%)	0	0 (0.0%)	1 (20.0%)	1 (6.7%)	1 (4.5%)	1 (2.4%)	0 (0.0%)	1 (1.8%)	0 (0.0%)	0 (0.0%)
25-50	0 (0.0%)	0	0 (0.0%)	0 (0.0%)	3 (20.0%)	5 (22.7%)	7 (31.8%)	7 (20.0%)	11 (20.0%)	8 (44.4%)	1 (10.0%)
50-75	0 (0.0%)	0	0 (0.0%)	1 (20.0%)	4 (26.7%)	7 (31.8%)	15 (35.7%)	9 (25.7%)	14 (25.5%)	3 (16.7%)	5 (50.0%)
75-100	0 (0.0%)	0	0 (0.0%)	1 (20.0%)	2 (13.3%)	2 (9.1%)	1 (2.4%)	2 (5.7%)	10 (18.2%)	3 (16.7%)	3 (30.0%)
Unsure	1 (100%)	0	1 (100%)	2 (40.0%)	5 (33.3%)	7 (31.8%)	18 (42.9%)	17 (48.6%)	19 (34.5%)	4 (22.2%)	1 (10.0%)
*p* = 0.270, Kendall’s tau = 0.086

**Table 6:** Association between opioid use and Numeric Pain Rating Scale score (*n* = 204)

	Opioid use	
	Yes *N* (%)	No *N* (%)
**Pain score**		
0	1 (0.8%)	0 (0.0%)
1	0	0
2	0 (0.0%)	1 (1.3%)
3	2 (1.6%)	3 (4.0%)
4	7 (5.4%)	8 (10.7%)
5	18 (14.0%)	4 (5.3%)
6	27 (20.9%)	15 (20.0%)
7	26 (20.2%)	9 (12.0%)
8	33 (25.6%)	22 (29.3%)
9	11 (8.5%)	7 (9.3%)
10	4 (3.1%)	6 (8.0%)
*p* = 0.505, Kendall’s tau = −0.043

**Table 7:** Associations between opioid use and concerns (*n* = 204)

	Opioid use	
	Yes *N* (%)	No *N* (%)
**Overdose**		
0	84 (65.1%)	52 (69.3%)
1	27 (20.9%)	8 (10.7%)
2	11 (8.5%)	9 (12.0%)
3	4 (3.1%)	6 (8.0%)
4	2 (1.6%)	0 (0.0%)
5	1 (0.8%)	0 (0.0%)
*p* = 0.813, Kendall’s tau = 0.016		
**Addiction**		
0	77 (59.7%)	44 (58.7%)
1	26 (20.2%)	13 (17.3%)
2	10 (7.8%)	10 (13.3%)
3	8 (6.2%)	6 (8.0%)
4	7 (5.4%)	1 (1.3%)
5	1 (0.8%)	1 (1.3%)
*p* = 0.864, Kendall’s tau = −0.011		
**Disapproval**		
0	95 (73.6%)	53 (70.7%)
1	14 (10.9%)	10 (13.3%)
2	8 (6.2%)	7 (9.3%)
3	4 (3.1%)	3 (4.0%)
4	7 (5.4%)	2 (2.7%)
5	1 (0.8%)	0 (0.0%)
*p* = 0.760, Kendall’s tau = −0.02		
**Affordability**		
0	8 (6.2%)	6 (8.0%)
1	10 (7.8%)	8 (10.7%)
2	24 (18.6%)	12 (16.0%)
3	37 (28.7%)	21 (28.0%)
4	30 (23.3%)	17 (22.7%)
5	20 (15.5%)	11 (14.7%)
*p* = 0.667, Kendall’s tau = 0.027		
**Side effects**		
0	37 (28.7%)	28 (33.7%)
1	31 (24.0%)	15 (20.0%)
2	18 (14.0%)	12 (16.0%)
3	26 (20.2%)	12 (16.0%)
4	11 (8.5%)	7 (9.3%)
5	6 (4.7%)	1 (1.3%)
*p* = 0.230, Kendall’s tau = 0.075		
**Driving**		
0	33 (25.6%)	20 (26.7%)
1	25 (19.4%)	15 (20.0%)
2	16 (12.4%)	10 (13.3%)
3	17 (13.2%)	14 (18.7%)
4	21 (16.3%)	12 (16.0%)
5	17 (13.2%)	4 (5.3%)
*p* = 0.426, Kendall’s tau = 0.048

**Table 8:** Associations between age and concerns for driving and disapproval of friends and family (*n* = 204)

	Age (years)						
Driving	25-30	31-40	41-50	51-60	61-70	71-80	81+
0	1 (50.0%)	5 (33.3%)	6 (15.8%)	20 (29.0%)	15 (27.3%)	5 (25.0%)	1 (20.0%)
1	0 (0.0%)	1 (6.7%)	13 (34.2%)	11 (15.9%)	9 (16.4%)	4 (20.0%)	2 (40.0%)
2	0 (0.0%)	2 (13.3%)	3 (7.9%)	12 (17.4%)	5 (9.1%)	4 (20.0%)	0 (0.0%)
3	1 (50.0%)	3 (20.0%)	5 (13.2%)	10 (14.5%)	10 (18.2%)	2 (10.0%)	0 (0.0%)
4	0 (0.0%)	4 (26.7%)	8 (21.1%)	8 (11.6%)	9 (16.4%)	4 (20.0%)	0 (0.0%)
5	0 (0.0%)	0 (0.0%)	3 (7.9%)	8 (11.6%)	7 (12.7%)	1 (5.0%)	2 (40.0%)
*p* = 0.833, Spearman’s rho = 0.015							
**Disapproval**	25-30	31-40	41-50	51-60	61-70	71-80	81+
0	2 (100%)	12 (80.0%)	25 (65.8%)	50 (72.5%)	36 (65.5%)	18 (90.0%)	5 (100%)
1	0 (0.0%)	1 (6.7%)	4 (10.5%)	7 (10.1%)	10 (18.2%)	2 (10.0%)	0 (0.0%)
2	0 (0.0%)	1 (6.7%)	4 (10.5%)	6 (8.7%)	4 (7.3%)	0 (0.0%)	0 (0.0%)
3	0 (0.0%)	1 (6.7%)	2 (5.3%)	1 (1.4%)	3 (5.5%)	0 (0.0%)	0 (0.0%)
4	0 (0.0%)	0 (0.0%)	3 (7.9%)	5 (7.2%)	1 (1.8%)	0 (0.0%)	0 (0.0%)
5	0 (0.0%)	0 (0.0%)	0 (0.0%)	0 (0.0%)	1 (1.8%)	0 (0.0%)	0 (0.0%)
*p* = 0.348, Spearman’s rho= −0.066							

## Abbreviations

ADHD: Attention deficit hyperactivity disorder
COVID-19: Coronavirus disease 2019
CP: Chronic pain
CPM: Center for pain medicine
DUIC: Driving under the influence of cannabis
MMIQ: Medical Marijuana Interest Questionnaire
MMJ: Medical marijuana
NPRS: Numeric pain rating scale
SSRI: Selective serotonin reuptake inhibitor
THC: Tetrahydrocannabinol
